# Effects of manual hyperinflation, clinical practice versus expert recommendation, on displacement of mucus simulant: A laboratory study

**DOI:** 10.1371/journal.pone.0191787

**Published:** 2018-02-12

**Authors:** Marcia S. Volpe, Juliane M. Naves, Gabriel G. Ribeiro, Gualberto Ruas, Mauro R. Tucci

**Affiliations:** 1 Department of Human Movement Sciences, Federal University of São Paulo, Santos, São Paulo, Brazil; 2 Department of Applied Physiotherapy, Federal University of Triângulo Mineiro, Uberaba, Minas Gerais, Brazil; 3 Laboratory for Medical Research 09, School of Medicine, University of São Paulo, São Paulo, Brazil; University of Notre Dame Australia, AUSTRALIA

## Abstract

**Introduction:**

Manual hyperinflation (MH), a maneuver applied in mechanically ventilated patients to facilitate secretion removal, has large variation in its performance. Effectiveness of MH is usually evaluated by its capacity to generate an expiratory flow bias. The aim of this study was to compare the effects of MH—and its resulting flow bias—applied according to clinical practice versus according to expert recommendation on mucus movement in a lung model simulating a mechanically ventilated patient.

**Methods:**

Twelve physiotherapists were asked to apply MH, using a self-inflating manual resuscitator, to a test lung as if to remove secretions under two conditions: according to their usual clinical practice (pre-instruction phase) and after verbal instruction to perform MH according to expert recommendation was given (post-instruction phase). Mucus simulant movement was measured with a photodensitometric technique. Peak inspiratory flow (PIF), peak inspiratory pressure (P_IP_), inspiratory time (T_INSP_), tidal volume (V_T_) and peak expiratory flow (PEF) were measured continuously.

**Results:**

It was found that MH performed post-instruction delivered a smaller V_T_ (643.1 ± 57.8 ml) at a lower P_IP_ (15.0 ± 1.5 cmH_2_O), lower PIF (38.0 ± 9.6 L/min), longer T_INSP_ (1.84 ±0.54 s) and lower PEF (65.4 ± 6.7L/min) compared to MH pre-instruction. In the pre-instruction phase, MH resulted in a mean PIF/PEF ratio of 1.73 ± 0.38 and mean PEF-PIF difference of -54.6 ± 28.3 L/min, both out of the range for secretion removal. In the post-instruction phase both indexes were in the adequate range. Consequently, the mucus simulant was moved outward when MH was applied according to expert recommendation and towards the test lung when it was applied according to clinical practice.

**Conclusions:**

Performance of MH during clinical practice with PIF higher than PEF was ineffective to clear secretion in a lung model simulating a mechanically ventilated patient. In order to remove secretion, MH should result in an adequate expiratory flow bias.

## Introduction

Manual hyperinflation (MH), which involves lung ventilation using a manual resuscitation bag, is a technique used in mechanically ventilated patients to assist with clearance of pulmonary secretions in addition to endotracheal suction. Although MH is widely used in Australia [1], the United Kingdom [2], the Netherlands [3], Brazil [4] and Sri Lanka [5] (and also recommended in reviews by authors from other nationalities [6–9]), scientific evidence supporting its efficacy on hard clinical outcomes is still lacking [10,11]. Use of MH has only been associated with short-term improvements in lung compliance, oxygenation and secretion clearance [10].

According to expert recommendation [11–14], MH should apply: 1) a larger than normal volume (up to 50% greater than the tidal volume delivered by the ventilator) with a slow inspiratory flow; 2) an inspiratory pause of 1–2 seconds; and 3) high expiratory flow. Effectiveness of MH is usually evaluated by its capacity to generate an expiratory flow bias (i.e., peak expiratory flow [PEF] higher than peak inspiratory flow [PIF]) which is believed to move secretions toward central airways through the two-phase gas liquid transport [15,16]. The expiratory flow bias is usually described as the ratio (PIF/PEF) or difference between the peak airflows (PEF-PIF). According to experimental studies, a PIF/PEF ratio lower than 0.9 [17,18] or a PEF-PIF difference higher than 17 L/min [19] is considered critical thresholds for the removal of lung secretions during mechanical ventilation. On the other hand, whenever the PIF exceeds the PEF, above those described thresholds, secretions may migrate deeper into the lungs. More recently, in an experimental study with mechanically ventilated pigs, in the semirecumbent position, a mean PEF-PIF difference of 33.0 ± 7.6 L/min was necessary to promote outward mucus clearance, while a mean PEF-PIF difference of 23.5 ± 8.6 L/min resulted in inward mucus transport [20]. Besides the fact that it was an *in vivo* experiment, one of the reasons that might explain why the expiratory bias flow threshold was higher than the previous one reported (17 L/min) is that mucus had to be transported against gravity since animals were in the 30-degree head-up position. Clearly the influence of airway flows on mucus movement during mechanical ventilation requires more investigation.

It has been suggested that MH may not have a standard practice worldwide and that it is usually applied with high PIF, which may result on insufficient expiratory flow bias or, worse, on an inspiratory flow bias (PIF > PEF) [8,14]. This fact might have contributed to the lack of studies showing that MH consistently affects broader clinical outcomes. Ortiz et al. have shown that their cohort frequently applied MH with two fast compressions of the resuscitator bag which resulted in high PIF and an inspiratory flow bias [21]. The authors explained that MH might have been customized in that way, because the generation of high PIF may stimulate patients’ cough, and consequently enhance secretion clearance, or at least enhance physiotherapists’ impression that it removes more secretions. However, the consequences might be the application of ineffective maneuvers, with an inspiratory flow bias, especially if the patient has a depressed cough reflex or inability to cough efficiently [19].

The aim of the present study was to compare the effects of MH—and its resulting flow bias—applied according to clinical practice versus according to expert recommendation on mucus movement in a lung model simulating a mechanically ventilated patient.

## Material and methods

This was a laboratory-based, crossover study, with assessor-blinded outcome analysis. The study was undertaken at a traditional laboratory at the Educational Center of Federal University of Triângulo Mineiro. Ethics committee approval for this study (N° 53938516.1.0000.5154) was provided by the institutional review board of Federal University of Triângulo Mineiro, Uberaba, MG, Brazil, and informed written consent was obtained from all participants.

### Participants

A convenience sample of 12 respiratory physiotherapists working at the Clinical Hospital of Federal University of Triângulo Mineiro, Uberaba, MG, Brazil, participated in the study. Recruitment was voluntary, unpaid, and comprised inclusion criteria of a minimum of 2 years of experience at working in intensive care unit (ICU). On the day of the study, physiotherapists working at the ICUs were approached by a researcher and were invited to participate in the study. Fourteen physiotherapists were on shift on the day of data collection, all of them were invited, but only 12 had a minimum of 2 years of experience at working in ICU and consented to participate in the study. After participating in the study, the physiotherapists were told to not comment about the study methodology with their colleagues.

### Interventions

**Fig 1:**
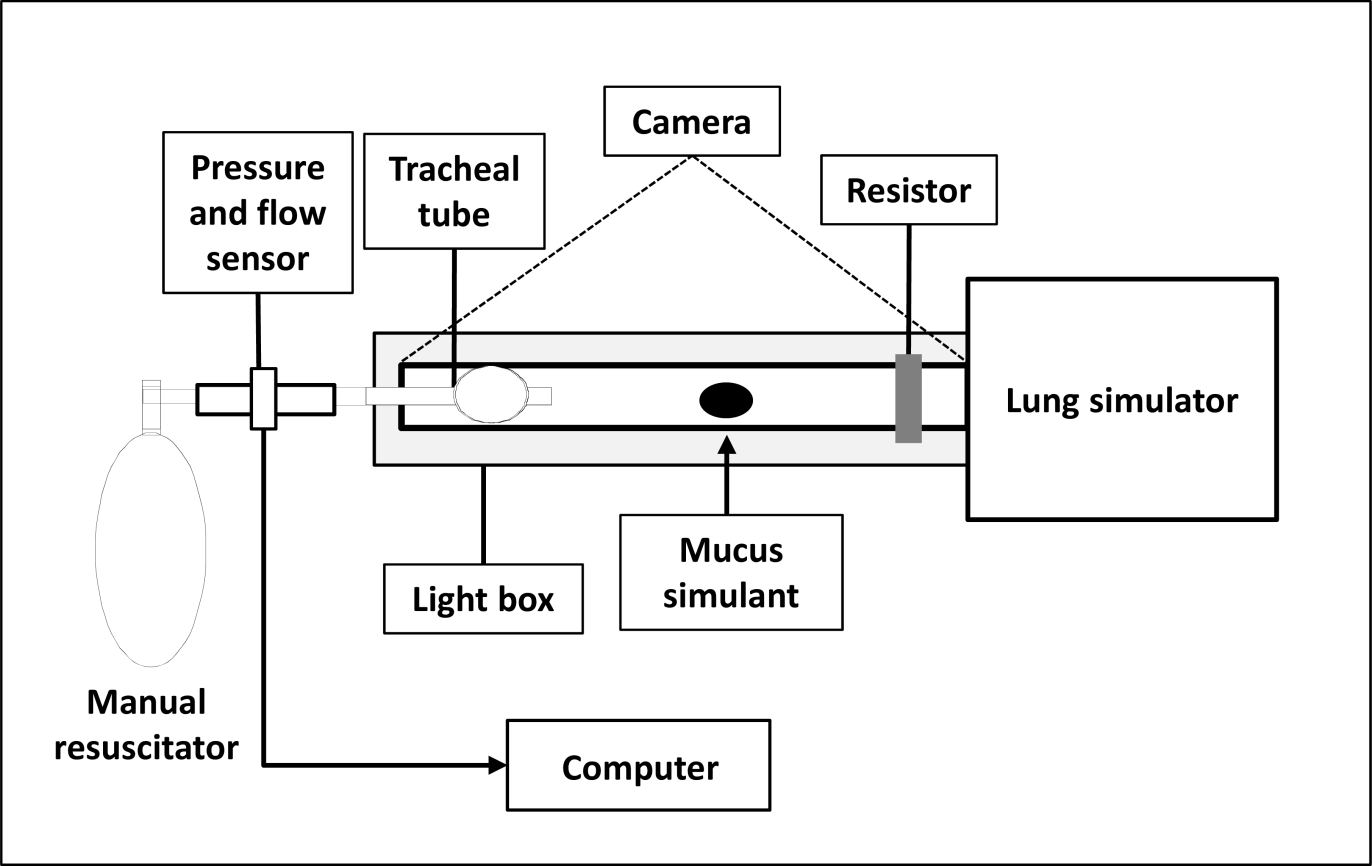
Experimental setup. Mucus simulant position was photographed with a 12 megapixel camera fixed 1.30 m above and perpendicular to the light box.

The study comprised two phases in which the displacement of mucus simulant was tracked following the application of MH performed by physiotherapists in order to assist with the removal of pulmonary secretion. Pre-instruction phase was conducted before and post-instruction phase after verbal instructions were given on how to apply MH, as explained below. The self-inflating manual resuscitator (Protec^®^, Cotia, São Paulo, Brazil) used had 1600 mL of capacity. The maneuver was applied into an artificial lung system (Intermed^®^, São Paulo, Brazil) via a transparent tubing (inner diameter 1.0 cm, length 30 cm, held horizontal on a light box). The test lung compliance and resistance were set at 0.05 L/cmH_2_O and 5 cmH_2_O/L/s, respectively simulating normal ventilated lungs without disease. Respiratory mechanics were measured proximally (between the tip of the transparent tubing and the self-inflating manual resuscitator) by the respiratory monitor CO_2_SMO^®^ Plus (Novametrix Medical Systems, Wallingford, CT). Fig 1 illustrates the experimental setup.

In the pre-instruction phase each physiotherapist was asked to perform five sequential MH breaths to assist pulmonary secretion clearance according to his/her usual clinical practice. In the post-instruction phase each physiotherapist was briefly and verbally instructed to perform MH according to expert recommendation [12,13]. The given instruction lasted approximately 2 minutes and was only verbal; the physiotherapists were not trained to follow a given pattern of insufflation. The verbal instruction was always performed by the same researcher, and it was given as follow: “Now you have to perform MH with a slow inflation, an inspiratory pause of 2 seconds, followed by rapid release of the bag”. While the instruction was being given the researcher demonstrated the maneuver with the manual resuscitator used in the study. The instruction was given twice. In both study phases before the physiotherapist started the maneuver, 1 mL of mucus simulant was injected into the center of the tubing and was allowed to settle for 3–5 min; thereafter an initial photograph was taken to register the mucus simulant initial position. After five breaths of MH, the maneuver was concluded and another photograph was taken. The photographs were analyzed offline to assess the MH effects on mucus movement. After each MH maneuver, the tube was washed, air-dried, and repositioned on the light box for the next experiment. The lung model was covered to not allow the physiotherapists to visualize mucus simulant displacement.

#### Mucus simulant

Synthetic solutions with standardized viscoelastic properties similar to human mucus were formulated, according to previously described methods [22]. Polyethylene oxide powder (1.5g) (Sentry Polyox WSR Coagulant, Dow Chemicals, Wilmington, DE) was dissolved in 100 mL of filtered water, at 100°C. The solution thicknesses at the concentration prepared (1.5%) simulated normal airway mucus. The mucus simulant was colored to allow quantitative photodensitometry, described below.

#### Mucus movement measurements

The transparent tubing (inner diameter 1 cm, length 30 cm) was positioned horizontally on a light box and mucus movement was photographed with a 12 megapixel camera fixed 1.30 m above and perpendicular to the light box. Photographs of mucus simulant position were obtained before and after five breaths of each performed MH. Image-analysis software (Sigmascan, Statistical Solutions, Saugus, MA) was used to evaluate mucus movement, by measuring the mucus area in number of pixels. A ruler was positioned next to the tubing to calibrate 1 pixel in centimeters. The image-analysis software can also measure the color intensity of each pixel in a measured object. The color intensity provides an indirect measure of mucus depth. Details of the technique used to measure mucus movement have been described elsewhere [19]. Mucus displacement after MH was evaluated based on the displacement of the center-of-mass. The image analysis software calculates the center-of-mass by determining a central location of the “object” after multiplying all pixels by their relative intensities.

#### Analysis of respiratory mechanics

Data acquired by CO_2_SMO^®^ Plus monitor were filtered and sampled at 100 Hz. Variable values for each MH breath were stored for subsequent analysis by attaching a computer running complementary software (Analysis Plus, Novametrix Medical Systems, Wallingford, CT) to the monitor. Four of the five MH breaths applied during each of the two study phases were selected and analyzed to obtain the values of peak inspiratory pressure (P_PI_), inspiratory time (T_INSP_), tidal volume (V_T_), PIF and PEF.

The primary outcome was PIF and PEF relationship (assessed by PIF/PEF ratio and PEF-PIF difference) and its influence on mucus simulant displacement.

#### Statistical analysis

The study was designed to have a 90% power to detect a difference in the PIF/PEF ratio between MH applied according to clinical practice (PIF/PEF ratio of 1.96, based on the study of Ortiz et al. [21]) and MH applied according to expert recommendation (PIF/PEF ratio of 0.90, based on the flow bias threshold described as necessary to remove mucus [17,18]), at a 2-sided α error of 5% for paired t test (standard deviation of difference was 0.99). This generated the sample size of 12 subjects using G Power 2 version 3.1.5.

Data were analyzed using SPSS version 20.0 (SPSS Inc. Chicago, Illinois, USA). The Shapiro-Wilk test showed that the tested variables had normal distribution and therefore data are presented as mean ± standard deviation. Differences between the two study phases (pre and post-instruction) were compared by paired samples t test. The level of significance was set at 0.05 for all tests.

## Results

The sample consisted of 12 physiotherapists (10 female, 2 male) aged 28 ± 4 years; ICU experience was 2 to 10 years, mean 5 ± 3 years. Four respiratory cycles from each of the two experimental conditions were analyzed for each one of the 12 physiotherapists, resulting in an analysis of 96 respiratory cycles.

### MH ventilation pattern during clinical practice versus expert recommendation

**Fig 2:**
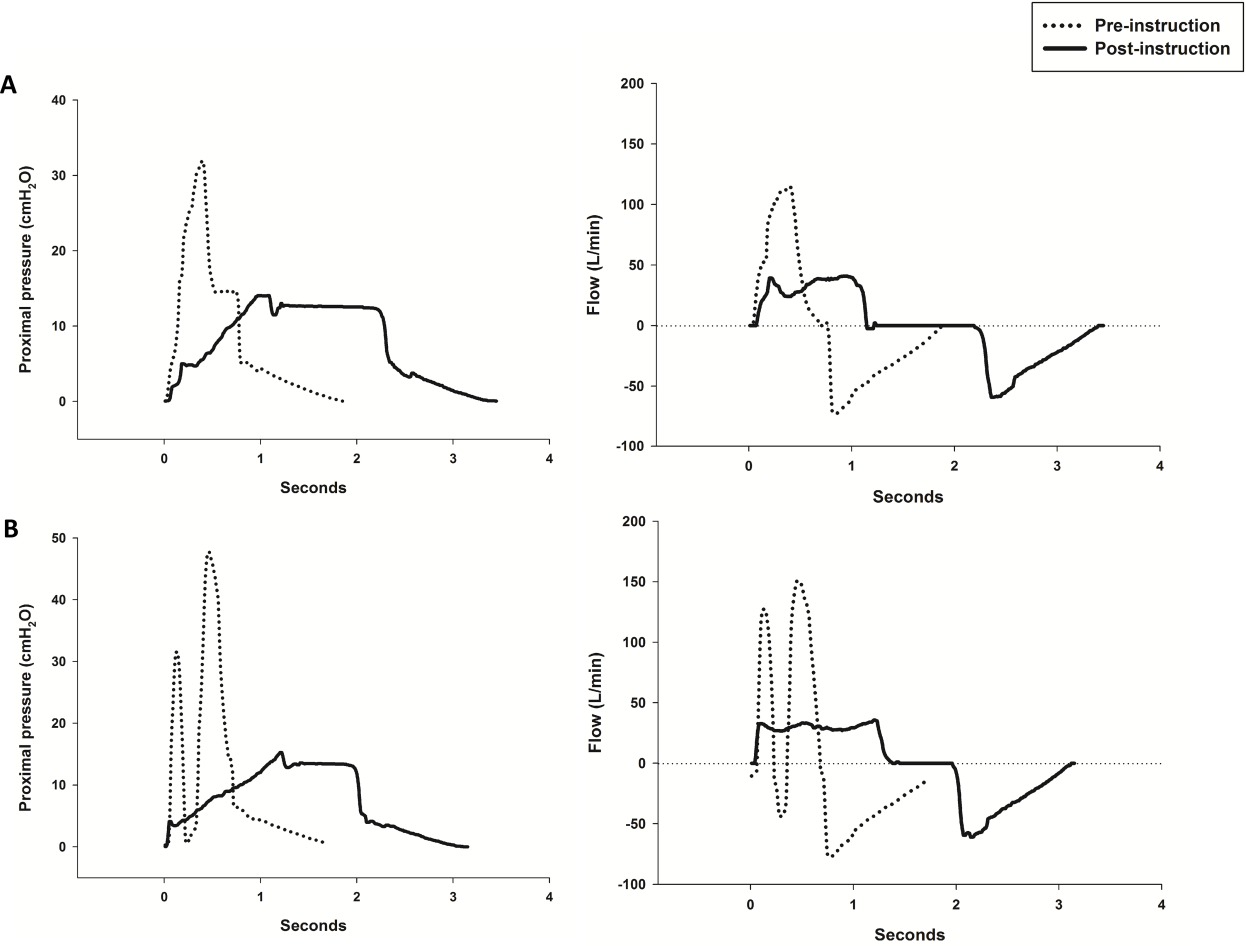
**Differences in proximal pressures (left column) and flows (right column) achieved during MH performance by two physiotherapists (A and B).** In the pre-instruction phase (dashed lines) MH was applied according to usual clinical practice and in post-instruction phase (solid black lines) MH was applied after receiving instruction to perform the maneuver according to expert recommendation.

The MH pattern applied according to usual clinical practice (pre-instruction) was very different from the MH applied post-instruction (Table 1, Fig 2). Five MH cycles—each cycle composed by 2 to 3 fast compressions of the resuscitator bag generating some level of breath stacking and without an inspiratory pause- were performed by seven physiotherapists (58.3%) during the pre-instruction phase (Fig 2B, S1 Fig). After instruction all physiotherapists improved the maneuver. Post-instruction MH delivered a smaller V_T_ at a lower P_IP_, lower PIF and longer T_INSP_ compared to pre-instruction (Table 1). As a result of a lower V_T_, PEF was also lower during post-instruction. Despite a lower PEF, the PEF-PIF difference and the PIF/PEF ratio resulted in an expiratory flow bias during the post-instruction phase while during the pre-instruction phase an inspiratory flow bias was created (Table 1).

**Table 1:** Mean (SD) for outcomes during MH for each study phase.

Variables	Pre-instruction *Clinical Practice*	Post-instruction *Expert recommendation*	*P value*
**V**_**T**_ (mL)	711.4 ± 76.1	643.1 ± 57.8	0.005
**T**_**INSP**_ (s)	0.62 ± 0.15	1.84 ± 0.54	< 0.001
**PIF** (L/min)	129.6 ± 28.8	38.0 ± 9.6	< 0.001
**PIP** (cmH_2_O)	39.1 ± 11.1	15.0 ± 1.5	< 0.001
**PEF** (L/min)	75.0 ± 5.2	65.4 ± 6.7	0.001
**PIF/PEF**	1.73 ± 0.38	0.58 ± 0.16	< 0.001
**PEF-PIF** (L/min)	-54.6 ± 28.3	27.5 ± 11.0	< 0.001
**CMD**^a^ (cm)	- 2.35 ± 0.63	0.52 ± 0.33	< 0.001

^a^A negative displacement indicates mucus movement towards the test-lung.

Abbreviations: CMD, center-of-mass displacement; PEF, peak expiratory flow; PIF, peak inspiratory flow; PIP, peak inspiratory pressure; T_INSP_, inspiratory time; V_T_, tidal volume.

### PIF and PEF relationship and its influence on mucus displacement

**Fig 3:**
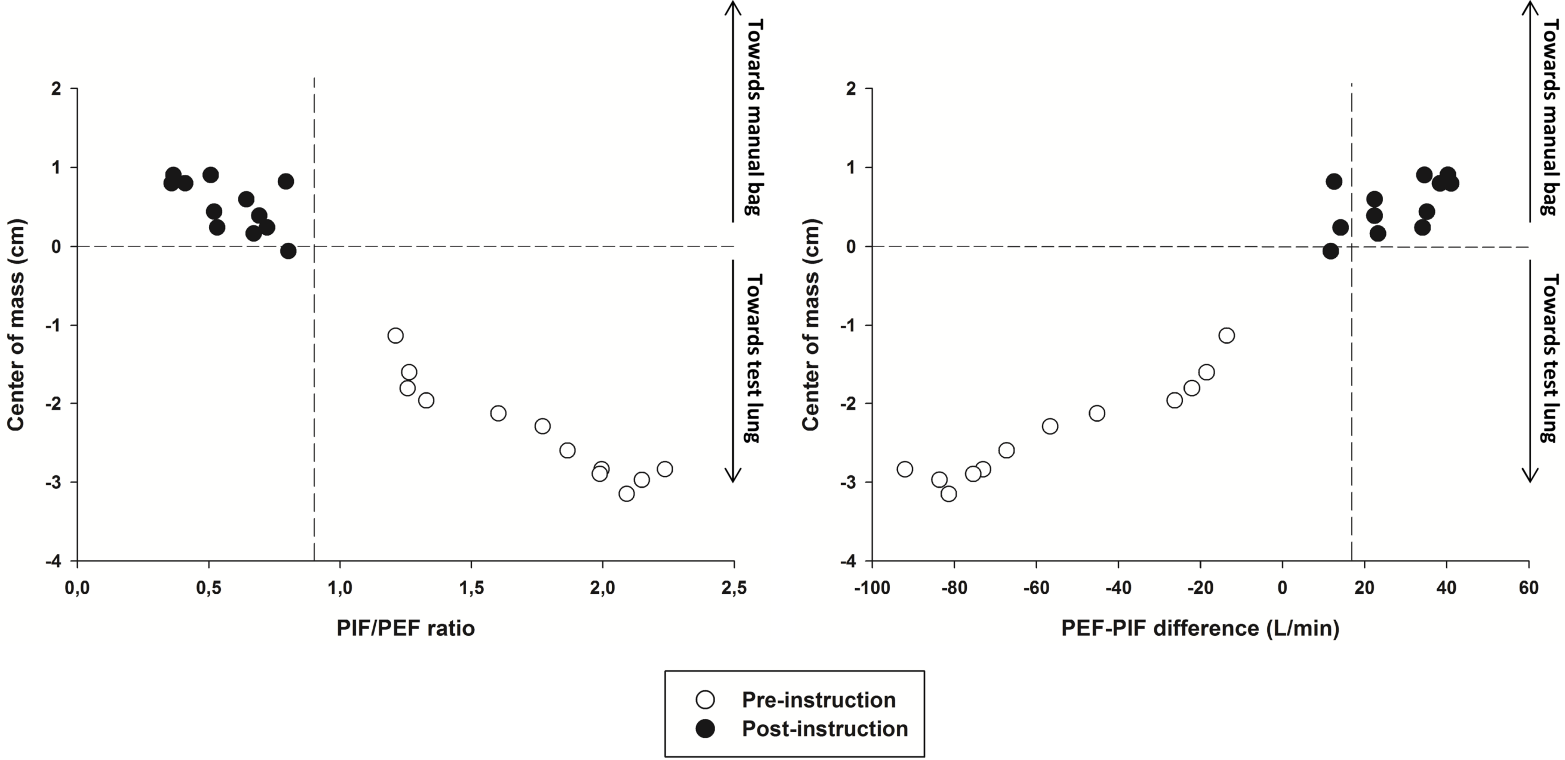
Relationship of center-of-mass displacement to PIF/PEF ratio and PEF-PIF difference obtained during the two study phases. A negative displacement indicates mucus movement towards the test lung. Dashed lines mark zero of center-of-mass displacement and the thresholds (PIF/PEF ratio < 0.9 and PEF-PIF difference > 17L/min) to facilitate comparisons between study phases. Note that only during the post-instruction phase (black circles) mucus simulant was moved outward. During the pre-instruction phase (white circles) mucus simulant was moved towards the test lung.

In the pre-instruction phase, that represented physiotherapists usual clinical practice, all PIFs were higher than PEFs. Therefore, MH resulted in a mean PIF/PEF ratio and mean PEF-PIF difference far distant from its respective described thresholds to move secretions toward the glottis (i.e. PIF/PEF< 0.9 and PEF-PIF difference >17 L/min) (Table 1). After instruction both thresholds were achieved as a consequence of a much lower generated PIF compared to pre-instruction. As a result, the center-of-mass of the mucus simulant was moved towards the test lung during MH performed according to usual clinical practice and towards the manual bag after instruction (Table 1, Fig 3).

Out of 24 applied MH only one (4%) did not present the expected mucus displacement according to PIF/PEF ratio, i.e. one maneuver resulted in a PIF/PEF ratio < 0.9 and did not result in mucus displacement towards the manual bag; according to PEF-PIF difference only two (8.3%) did not result in the expected direction displacement (Fig 3).

## Discussion

Results of this investigation affirm that MH should be performed according to expert recommendation in order to enhance clearance of secretions from the airways. Moreover, it is important to notice that the relationship established between PIF and PEF was the key-variable to facilitate secretion clearance and not a high PEF alone. The thresholds described in the literature to remove pulmonary secretion, PIF/PEF ratio < 0.9 and PEF-PIF > 17L/min, were consistent with current results. Out of 24 applied MH, only one to two maneuvers (4–8.3%) did not result in the expected mucus displacement direction.

To the best of the authors’ knowledge, this is the first study that shows in a lung model simulating a mechanically ventilated patient without lung disease that during MH an expiratory flow bias is necessary to remove mucus. This finding is in agreement with a previous study that demonstrated in a glass tube model that MH by generating higher PEFs was superior to mechanical ventilation for effective secretion clearance [23]. Current results also suggest that MH with an inspiratory flow bias may move secretion deeper into the lungs—at least if the patient is heavily sedated and without a preserved cough reflex.

Additionally, this study is in accordance with those of Ortiz et al., in which most of the physiotherapists performed MH, before verbal instruction, with high PIF and short T_INSP_ [21]. Two hypotheses may explain this finding: 1) physiotherapists education on MH did not emphasize the need for an expiratory flow bias, and/or 2) they have customized the maneuver along their clinical practice according to the impression that applying high PIF (with two to three fast bag-compressions generating some level of breath stacking) stimulates cough and enhances clearance. As this study used a lung model, conclusions regarding the influence of high PIF in stimulating cough and augmenting secretion removal were not possible; this warrants further investigation. However, it is important to notice that the mean PIF applied in the pre-instruction phase (129.6 L/min) was almost two times higher than the median cough peak flow of 70 L/min found in a recently published cohort that evaluated 356 patients on mechanical ventilation with planned extubation [24]. This suggests that MH applied in usual clinical practice at our facility may even be ineffective (or potentially deleterious) in critically ill patients with a preserved cough reflex. Reinforcing this concerning scenario, a cough peak flow of 60 L/min has been reported as a predictor of extubation success in critically ill mechanically ventilated patients [25–27].

Interestingly, although larger V_T_s appear beneficial for achieving an expiratory flow bias, in the current study the delivered V_T_ was lower than that usually reported in other studies, but MH performed post-instruction could still generate an adequate expiratory flow bias. The lower V_T_ might be explained by the type of manual resuscitator used which had a self-inflating bag. This type of resuscitator, which is more frequently used in Brazil, usually generates lower V_T_ than the resuscitators that have flow-inflating bags [28–30]. This difference might be related to the size of the bag employed: 1.6 L in the present study, compared with 2.0 liters in the other studies [28–30]. Moreover, the shorter V_T_ delivered in this study (mean of 643 mL) might explain why a T_INSP_ shorter than two seconds (mean of 1.84 s) was able to generate an adequate expiratory flow bias. A recent bench study demonstrated that, to achieve sufficient expiratory flow bias during MH, it was necessary to have a T_INSP_ of at least three seconds with normal compliance lungs and two seconds with lower compliance lungs [31]. However, in their study the delivered V_T_ was standardized in 1.4 L which, clearly, required a longer T_INSP_ to generate a lower PIF.

Of note, in the pre-instruction phase the PIP exceeded the traditional limit of 40 cmH_2_O (mean PIP was 39.1 cmH_2_O) during 6 of 24 applied MH maneuvers, which may cause some concern as this limit is set to prevent barotrauma [32]. However, it has been demonstrated that during MH, alveolar pressures, roughly represented by plateau pressures, are usually within the safe range despite of high PIP [21]. As plateau pressures correlate better with barotrauma that PIP, it is unlikely that the performed MH would have caused barotrauma [33].

It is worth mentioning that high PIP could have been avoided if a pressure manometer was used with the MH circuit [34]. However, this tool is not frequently applied in ICUs in Brazil, and its use in this study would have interfered on reproducing MH as it is actually performed in usual clinical practice at our facility.

In this study, education had a significant impact on the ability to remove the mucus simulant. These results highlight the importance of training programs that teach physiotherapists how to correctly apply MH according to expert recommendations which include the goals of achieving an expiratory flow bias and limiting PIP.

This study has a few limitations. First of all, the use of a lung model produced inherent limitations, which requires caution to extrapolate results to a clinical population. Second, the simulated mucus, although previously used by others [19,22,23], is not equivalent to real mucus, which differs and varies markedly in consistency and in composition and among patients. Third, the behavior of the mucus simulant in a biologically branched network was not investigated. Fourth, gravity which plays an important role on secretion movement in the airways was not taken in consideration [35]. Fifth the results are based on small sample size. However, this small sample of physiotherapists was used to obtain a qualitative analysis of how MH is usually performed by experienced professionals at our facility and not to address how MH is performed more widely across Brazil or elsewhere.

In conclusion, MH applied in usual clinical practice at our facility, with high PIFs, was ineffective to clear mucus clearance in a lung model simulating a mechanically ventilated patient without lung disease. In order to remove secretions, MH should be performed with a slow insufflation to result in an adequate expiratory flow bias.

## Supporting information

S1 File Study data. (XLSX)

S1 Fig Flow, pressure and volume tracings of the five MH breaths applied by one of the physiotherapists according to his usual clinical practice. (TIF)
